# Experimental investigation of jet-induced wall pressure fluctuations over a tangential flat plate at two Reynolds numbers

**DOI:** 10.1038/s41598-020-66037-2

**Published:** 2020-06-04

**Authors:** Stefano Meloni, Alessandro Di Marco, Matteo Mancinelli, Roberto Camussi

**Affiliations:** 1 Department of engineering, University of RomaTre, Rome, Italy; 2 Département Fluides, Thermique et Combustion, Institut Pprime - CNRS-Université de Poitiers-ENSMA, Chasseneuil-du-Poitou, Poitiers, France

**Keywords:** Environmental sciences, Engineering, Physics

## Abstract

The wall pressure fluctuations induced by a subsonic circular jet on a rigid flat plate have been investigated considering two jets with different exit section diameters at the same Mach number. The analysis is aimed at completing the series of papers presented by the authors on the interaction between a subsonic jet and infinite tangential flat plate where the exit Mach number was the only parameter of the jet flow that was varied. In order to analyse other effects out of the Mach number, two configurations with different nozzle exhaust diameters were explored with the objective of isolating the Reynolds number effect keeping fixed the exit Mach number. The nozzle exhaust diameters are 12 *mm* and 25.4 *mm* and the instrumented flat plate, installed parallel to the jet flow, is moved at different radial distances from the jet axis. The pressure footprint on the plate has been measured in the stream-wise direction by means of a pair of flush-mounted pressure transducers, providing point-wise pressure signals. Wall pressure fluctuations have been characterised in terms of spectral and statistical quantities. The effect of Reynolds is evidenced and possible scaling relationships that account for the Reynolds dependence are proposed. Implications for modeling the spectral coherence have been considered by the application of the Corcos’ model and the effect of the jet Reynolds number on the model coefficients is analyzed.

## Introduction

The noise emitted by the jet turbulent structures is a long-standing issue which has received particular attention in the last 60 years by the scientific and industrial communities. As a matter of fact, when the jet exhausting turbulent flow interacts with the atmosphere it produces a considerable noise, called mixing noise^1^. In the last century, an analogy between the full non linear acoustic flow problem and the in-homogeneous acoustic wave equation was provided by Lighthill^2^ forming the basis of the jet aeroacoustics. The Lighthill’s analytical formulation was developed for isolated jets and represented a robust path-line for researchers and manufacturers to develop models able to predict the noise generated by compressible jets.

Nowadays, in order to reduce fuel consumption, air pollution and noise emissions, manufactures are moving towards the production of Ultra High Bypass Ratio (UHBPR) turbofan engines, this architecture comprising a reduction of the nozzle exhaust velocity, which is a benefit for the noise emissions, and an increase of the fan diameter to the purpose of maintaining the same level of thrust.

Being fixed the clearance available for the engine installation, the increasing size of the nozzle leads to a more coupled jet-wing architecture, and brings complex interaction phenomena between the jet and the airframe surfaces. From the viewpoint of the noise emission, the behavior is much more complex with respect to the case of the standard isolated jet studied so far. The rigid surface modifies the jet plume, alters the mixing noise sources and affects the way acoustic waves propagate in the far field. Moreover, considering long range aircrafts with a four-engines configuration, the interaction between the flow exhausting from engines closest to the fuselage and aircraft body is probably an important component of the interior noise.

The additional noise and pressure load originated by the jet-surfaces interactions motivated the interest towards installation effects of both industries and researchers. As shown in the literature, installation effects generated by the interaction between different aircrafts noise sources have been investigated in several works (see e.g.^3–6^). Due to the difficulties in investigating the jet-surface interaction phenomena in a full-scale configurations^5^, several studies have been carried out on simplified arrangements^3^. By mocking up the aircraft structures with a tangential flat plate, shielding/scattering effects have been investigated (e.g.^7,8^) and the near- and far-field pressure statistics have been measured^9^. More complex wing-flap configurations have been analyzed by Jordan *et al*.^4^ shedding light on resonance mechanisms originating acoustic tones also investigated in some free jet configurations^10–12^. The jet-induced wall pressure field is of interest for predicting the interior noise generation and the vibro-acoustic response of the aircraft surfaces. This aspect has been the subject of a series of investigations performed by the authors on a simplified geometry in incompressible and compressible flow conditions (see^13–15^).

Despite the large body of parametric studies carried out on jet-plate arrangements, the effect of the jet Reynolds number (*Re*) has not been analyzed so far. As pointed out in^16^, the noise induced by an exhaust flow is a researching challenge since each turbulent jet is very sensitive to its own initial conditions. This issue motivated several studies carried out in the past on free jets by varying the shape of the nozzle exhaust and the jet *Re* (e.g. among many^17,18^) but similar investigations in the case of jet installed configurations are not present in literature.

The objective of the present work is the investigation of the effect of *Re* on the wall pressure fluctuations induced by a weakly compressible jet overflowing tangentially a rigid flat plate. Performing experiments at different *Re* maintaining constant the Mach number is a challenging task. In the present approach, the *Re*, is varied by installing two nozzles with different exhaust diameter: *D*_1_ = 12 *mm* to which corresponds ${\mathrm{Re}}_{{D}_{1}}=1.47\cdot {10}^{5}$ and *D*_1_ = 25.4 *mm* to which corresponds ${\mathrm{Re}}_{{D}_{2}}=3.12\cdot {10}^{5}$. It must be taken into account that due to limitations related to the experimental approach, a larger variation of *Re* was not achievable. Nonetheless, this analysis provides, for the first time, the basis for a physical understanding of the Reynolds number effects on the jet-induced wall pressure fluctuations. This is the reason because of the analysis has been circumspect to two Reynolds very different but having the same order of magnitude. Though this analysis puts, for the first time, basis for the physical understanding of the effects induced by the nozzle exhaust Reynolds number on wall pressure fluctuations. The jet Mach number was set equal to *M*_*j*_ = 0.5 and the wall pressure fluctuations were measured for two radial distances of the flat plate from the nozzle axis, *H*/*D* = 0.75 and *H*/*D* = 2. It should be pointed out that, as reported in^13,15,19,20^, the jet-plate distance is a driving parameter of the physics of the jet-surface interaction. Specifically, for large jet-plate distances the plate effect on the aerodynamic field is expected to be weak^19,21,22^ as well as the jet footprint on the surface. On the contrary, for the closest-coupled configuration (H/D = 0.75) the jet-surface interaction phenomena are more significant leading to the development of a turbulent boundary layer (TBL)-like zone on the plate far downstream of the nozzle exhaust.

Measurements were carried out by flush-mounted wall pressure transducers, positioned in stream-wise direction from *x*/*D* = 1 to *x*/*D* = 25. The data were analyzed in terms of single-point and two-point statistics and implications for spectral modeling of both auto-spectra and coherence functions are discussed.

The paper is organised as follows: the experimental setup is reported in section 2; section 3 illustrates the main results on the statistical and spectral characterization of the wall pressure fluctuation field; conclusions are provided in section 4.

## Experimental setup

**Figure 1:**
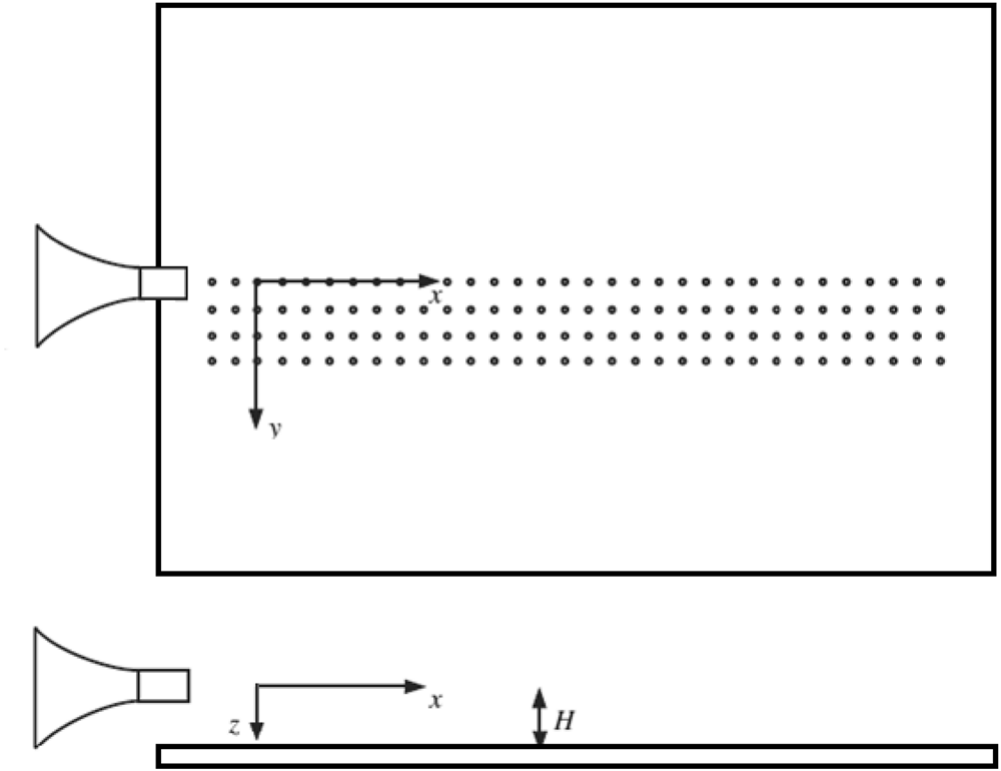
Sketch of the experimental setup.

Experiments have been carried out in the semi-anechoic chamber available in the laboratory of fluid-dynamics ‘G.Guj’ in the Department of Engineering of ‘Università degli Studi Roma Tre’. The size of the chamber is 2 m*4 m*3 *m* and the sound absorbing panels provide fully anechoic conditions for frequencies above 500 *Hz*. The feed line consists in a compressed dry air duct supplied by a 2 *m*^3^ tank at 8 bar connected to a compressor. Honeycomb panels and turbulence grids are installed in the pipe in order to have the desired flow quality. The velocity is regulated by controlling the pressure of the incoming flow with an electronically-driven valve. The flow conditions at the nozzle inlet are continuously monitored by a thermocouple and a pressure transducer. The knowledge of pressure and temperature at the inlet section of the nozzle allowed us to analytically determine the jet Mach number (*M*_*j*_) using isentropic relations, more details about the experimental facility are reported in^23^. In order to study the installation effects a rigid flat plate was installed parallel to the nozzle axis. A sketch of the experimental setup is represented in Fig. 1.

The coordinate system, is chosen with the origin at the jet exit, the x-axis being in the stream-wise direction, the span-wise direction is y whereas z is the crosswise coordinate perpendicular to the plate, as reported in Fig. 1. Two different test cases are analyzed corresponding to two nozzle exhaust diameters: *D*_1_ = 12 *mm* and *D*_1_ = 25.4 *mm*. The flat plate was installed at two radial distances from the nozzle axis: *H*/*D* = 0.75 and *H*/*D* = 2. The plate is 0.8 m wide and 1.1 *m* length, this dimension range allows us to consider the plate infinite. The plate leading edge is positioned upstream of the jet nozzle exit avoiding any acoustic effect generated by the flow over-passing the leading edge. The side-edge and trailing-edge of the plate are positioned very far from the nozzle exhaust, thus their interaction with the jet flow field is considered negligible. Four rows of 50 holes with a separation distance between two consecutive pressure taps of 12 *mm* were realised on the surface. A couple of pressure transducers (Kulite-Mic190M), used also in^19^, were flush-mounted to measure the wall pressure fluctuations on the flat plate. The pressure transducers have a diameter equal to 3.8 *mm* that fits the pressure tap and a frequency response up to 100 *kHz*. The holes not used for the measurements were covered using adhesive tape to avoid resonant effects. Data were acquired setting the cut-off filter of the Kulite signal conditioner depending on the expected turbulence scales of interest. Specifically, for the 25.4 *mm* nozzle the cut-off filter was set at 30 *kHz* and the sampling frequency at 100 *kHz*, whereas for the 12 *mm* nozzle the cut-off was set at 70 *kHz* and the sampling frequency at 200 *kHz*. The bandwidth of the pressure transducers were extended using a resonance compensator set at 32 *kHz*. Pressure taps not involved in the measurement were covered to avoid spurious effects on the acquired signals. The pressure signals were acquired by a Yokogawa digital scope (DL780E).

## Results

### Single point statistics

In order to characterize the load induced by the wall pressure field on the surface, the overall sound pressure level (*OASPL*)^24^ and its normalized counterpart (OASPL^*^) were computed. The two quantities are defined as follows:

1 $$OASPL=10{\log }_{10}\left(\frac{{\sigma }_{p}^{2}}{{P}_{ref}^{2}}\right)$$

2 $$OASP{L}^{\ast }=10{\log }_{10}\left(\frac{{\sigma }_{p}^{2}}{{P}_{ref}^{2}\cdot {\mathrm{Re}}_{D}^{k}}\right)$$

where *σ*_*p*_ is the standard deviation of the pressure signal, *P*_*ref*_ is the reference sound pressure in the air (20 *μPa*)^24^, *Re*_*D*_ is the nozzle exhaust Reynolds number and *k* is the exponent of the Reynolds number that depends on the jet-surface interaction relevance, and thus on the jet-plate interaction zones (see e.g.^13,21^). The nozzle exhaust Reynolds number, *Re*_*D*_, has been evaluated as follows:

3 $${{Re}}_{D}=\frac{\rho {U}_{j}D}{\mu }$$

where *U*_*j*_ is the nozzle exhaust velocity, *ρ* is the flow density and *μ* is the flow viscosity.

**Figure 2:**
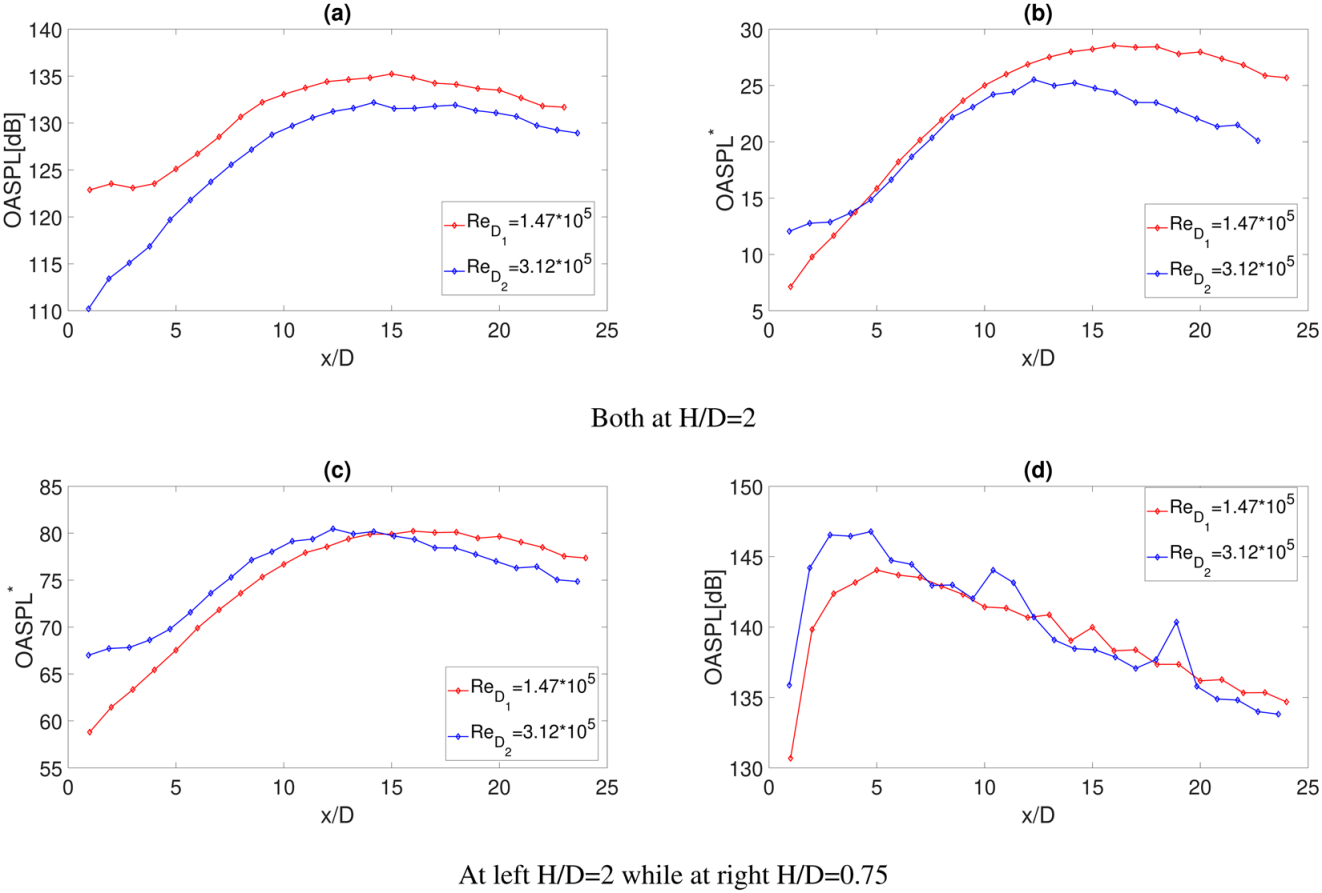
Stream-wise trends: (**a**) *OASPL* with the plate at *H*/*D* = 2. (**b**) OASPL^*^ with *k* = 2 and the plate at *H*/*D* = 2. (**c**) OASPL^*^ with *k* = 1 and the plate at *H*/*D* = 2. (**d**) *OASPL* with the plate at *H*/*D* = 0.75.

The *OASPL*s are reported in Fig. 2. At *H*/*D* = 2 (Fig. 2a), consistently with the results reported in the literature (e.g.^13,19,20^), the *OASPL* trend is strongly dependent on the stream-wise location. For small *x*/*D* the *OASPL* increases and reaches its maximum at the jet impact point on the plate; It remains about constant immediately downstream of it and decreases for large *x*/*D* where a TBL-like behaviour is reached. According to e.g.^13,19,21^, three jet-surface interaction zones can be distinguished: in the first zone, at low *x*/*D*, the jet has not yet impinged on the plate; the second zone corresponds to the region where the jet starts to interact with the plate downstream of the impact point; in the third zone, at large *x*/*D*, a TBL-like behaviour is found. The velocity field as well as the jet impact point have been evaluated in previous works on the same configuration and Mach number (see e.g.^13,19,21^). It has been found the jet impact point is at about *x*/*D* = 8 for the plate positioned at *H*/*D* = 2 and at about *x*/*D* = 4 for *H*/*D* = 0.75, and it was demonstrated that downstream of it, the OASPL remains about constant. As it is possible to observe in Fig. 2(a,d), the jet impact point is only very slightly influenced by different nozzle exhaust Reynolds numbers. The scaled OASPL^*^ trends for values of the *k* exponent equal to 2 and 1 are reported in Fig. 2(b,c), respectively. We note that the *OASPL* collapse is verified in the first and in the second jet-surface interaction zones for values of the k exponent equal to 2 and 1, respectively. The quadratic dependence of the *OASPL* on the jet Reynolds number can be explained by the fact that in the first zone, where no interaction occurs between the jet and the surface, the pressure fluctuations intensity is only dependent on the jet mass flow rate, which in turn depends on the nozzle exhaust section. This interpretation agrees with the Lighthill’s analogy that prescribes a quadratic dependence of the noise intensity on the nozzle exhaust diameter. On the contrary, for 10 < *x*/*D* < 20 where the jet starts to interact with the surface, we observe a smaller gap, between the two OASPL trends, using an exponential value of the Re number close to 1. A very different behaviour of the OASPL trends is detected at *H*/*D* = 0.75 with respect to the one detected at *H*/*D* = 2. The rapid increase at small axial positions is probably due to the jet shear layer and to the impact of the flow on the rigid surface. The OASPL decrease observed downstream of x/D = 6 could be likely related to the development of a TBL-like zone. As reported in Mancinelli *et al*.^14^ the development of the jet shear layer is prevented by the presence of the plate. The lack of entrainment induces a radial velocity component responsible of the observed Coanda effect and a slight anticipation of the jet impact point for small *H*/*D*. The OASPL analysis clarifies that the wall pressure fluctuations depend on the nozzle exhaust Reynolds number in a different way according to each jet-plate interaction zone. We finally point out that a collapse of the OASPL using only the measured data is detected, thus without any scaling factor.

**Figure 3:**
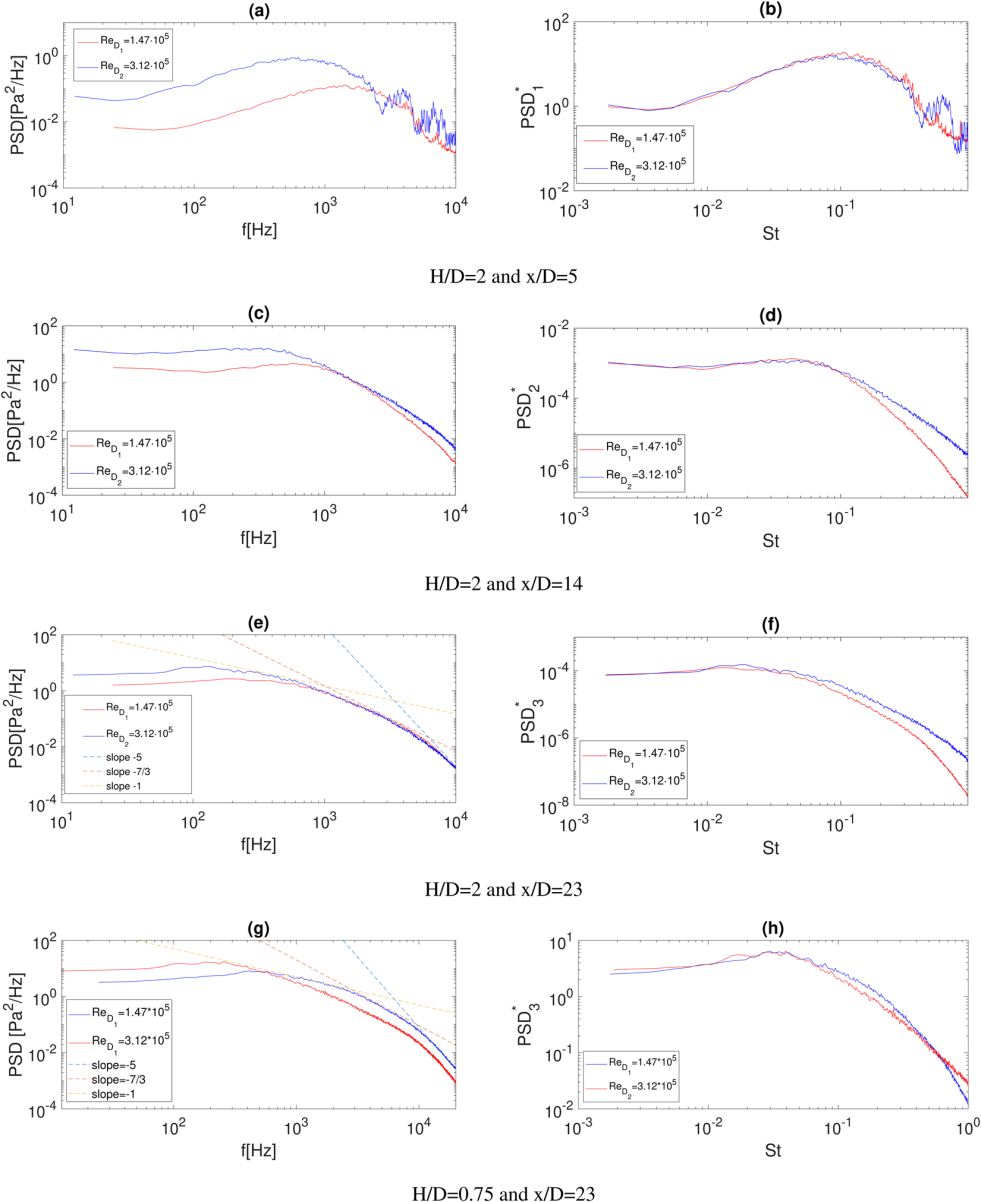
*PSD*s at different axial positions and different Reynolds numbers: (**a**) *H*/*D* = 2 and *x*/*D* = 5; (**c**) *H*/*D* = 2 and *x*/*D* = 14; (**e**) *H*/*D* = 2 and *x*/*D* = 23; (**g**) *H*/*D* = 0.75 and *x*/*D* = 23. Non dimensional spectra (PSD^*^) at the same positions: (**b**) *H*/*D* = 2 and *x*/*D* = 5; (**d**) *H*/*D* = 2 and *x*/*D* = 14; (**f**) *H*/*D* = 2 and *x*/*D* = 23; (**h**) *H*/*D* = 0.75 and *x*/*D* = 23. Dashed lines represent the typical decay laws encountered in fully developed TBL.

The uni-variate statistics were characterized in the frequency domain through the power spectral density (*PSD*) evaluated by means of the Welch’s method^25^ and results are reported in Fig. 3. It is observed that the shape of the spectrum does not change with the Reynolds number but it is shifted in the frequency domain according to the different Strouhal numbers associated with the different nozzle exhaust diameters. Scaling laws were evaluated to model the dependence of the auto-spectra on the Reynolds number. Different scaling factors suitable for the three different jet-plate interaction zones are reported in the following equations:

4 $$PS{D}_{1}^{\ast }=\frac{PSD}{{q}^{2}}\cdot \frac{x}{D}\cdot \frac{{U}_{j}}{D}\cdot \frac{{A}_{i}}{{A}_{j}},$$

5 $$PS{D}_{2}^{\ast }=\frac{PSD}{{q}^{2}}\cdot \frac{x}{D}\cdot \frac{{U}_{j}}{D}\cdot {\left(\frac{{A}_{i}}{{A}_{j}}\right)}^{\frac{1}{2}},$$

6 $$PS{D}_{3}^{\ast }=\frac{PSD\cdot {U}_{j}}{{q}^{2}\cdot D},$$

$\frac{{A}_{i}}{{A}_{j}}$ is the nozzle contraction area ratio where *A*_*i*_ the nozzle inlet area and *A*_*j*_ is the nozzle exhaust area, thus related to the square of the nozzle diameters. Instead *q* is the dynamic pressure evaluated as follows:

7 $$q=\frac{1}{2}\gamma {M}_{j}^{2}{P}_{amb}$$

where *γ* is the ratio of specific heats, *M*_*j*_ is the nozzle exhaust jet Mach number and *P*_*amb*_ is the ambient pressure.

In the first region, where pressure is induced by a free-shear-layer, the scaling criterion is the one reported in Eq. (4). It is similar to the scaling procedure adopted for free jets^2,26^ and, as shown in Fig. 3(a,b), provides a quite good collapse of the spectra. In the region close to the jet impact point, the dependence upon the jet *Re* number weakens and, as shown in Fig. 3(c,d), the scaling criterion of Eq. (5) provides a good collapse of the spectra in the low frequency range. In the farthest region, Fig. 3(e), a boundary layer is formed on the plate and the spectra exhibit power decay laws typical of fully developed *TBL*s. Indeed, a slope ∝ *f* − 1 is found in the frequency range close to the maximum of the *PSD* and, for higher frequencies, a power decay law ∝ *f* − 7/3 is observed. In the very high-frequency range, the spectral shape exhibit a power law decay close to ∝ *f* − 5 as an effect of the viscous dissipation^27^.

The effect of *Re* is negligible at high frequency whereas a discrepancy remains at lower frequencies. The scaling factor of Eq. (6) is based on classical models for wall pressure turbulent boundary layers^28^ and, as shown in Fig. 3(f), leads the low-frequency region of the spectra to collapse well.

Dimensional and scaled PSDs for the jet-plate distance *H*/*D* = 0.75 and *x*/*D* = 23 are eventually reported in Fig. 3(g,h). For the smallest *H*/*D* the jet impacts the plate much upstream and thus a turbulent boundary layer develops over a larger streamwise region. Therefore, the decay laws *f*^−5^ and *f*^−1^ associated with the classical boundary layer behavior are more pronounced with respect to the case at *H*/*D* = 2. Also for *H*/*D* = 0.75 it is observed that a good collapse of the spectra is achieved using Eq. (6) (Fig. 3h).

**Figure 4:**
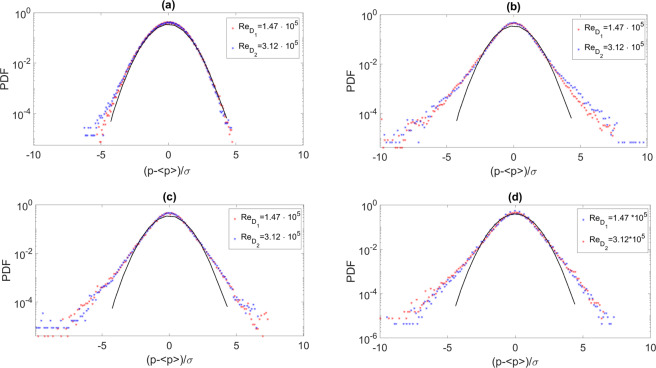
Axial evolution of the pressure PDF for different Reynolds number and different positions. (**a**) *x*/*D* = 5 and *H*/*D* = 2. (**b**) *x*/*D* = 14 and *H*/*D* = 2. (**c**) *x*/*D* = 23 and *H*/*D* = 2. (**d**) *x*/*D* = 23 and *H*/*D* = 0.75. Markers are for experimental *PDF*s and the black continuous line is for the reference Gaussian distribution.

Further insights into the uni-variate statistics are achieved through the estimations of the probability density functions (*PDF*) and the computation of the third and fourth order moments (skewness and kurtosis respectively), defined as:

8 $$s=\frac{E{(p- < p > )}^{3}}{{\sigma }_{p}^{3}}$$

9 $$k=\frac{E{(p- < p > )}^{4}}{{\sigma }_{p}^{4}}$$

where <*p*> is the mean of the signal p and *E*() is the expected value. The *PDF*s are presented in reduced form, i.e. normalized in order to have zero mean value and unitary standard deviation. Experimental *PDF*s are compared with a reference Gaussian distribution and are reported in Fig. 4.

**Figure 5:**
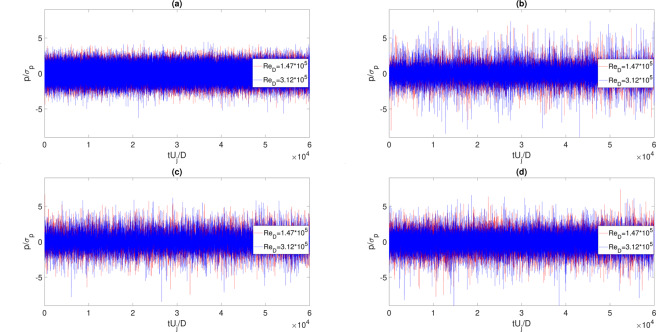
Pressure signals time evolution for different Reynolds numbers and different axial positions: (**a**) *x*/*D* = 5 and *H*/*D* = 2. (**b**) *x*/*D* = 14 and *H*/*D* = 2. (**c**) *x*/*D* = 23 and *H*/*D* = 2. (**d**) *x*/*D* = 23 and *H*/*D* = 0.75.

**Figure 6:**
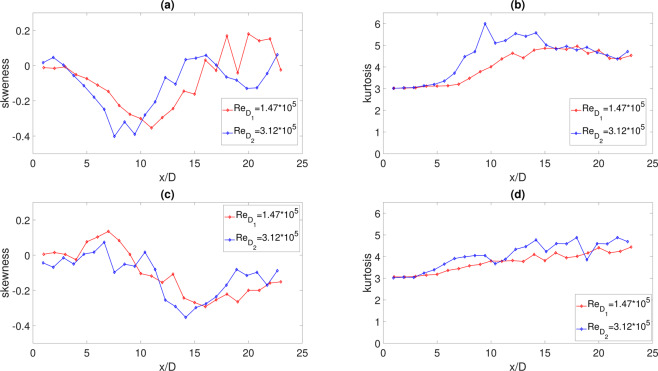
Axial evolution of the skewness and kurtosis factors at different Reynolds numbers: (**a**) skewness at *H*/*D* = 2. (**b**) kurtosis at *H*/*D* = 2. (**c**) skewness at *H*/*D* = 0.75. (**d**) kurtosis at *H*/*D* = 0.75.

We observe that, independently of *H*/*D* and *Re*, downstream of the impact point, the *PDF*s are strongly non Gaussian. They exhibit quasi–exponential tails that are traces of the intermittent statistic typical of turbulent flows. On the other hand, the constant values observed for amplitudes much smaller or much larger than the mean value are due to a lack of statistical convergence. This behavior is confirmed by the skewness and flatness factors reported in Fig. 6. A region where the skewness factor is negative is found close to the impact zone for both the jet Reynolds numbers and both *H*/*D* (Fig. 6a,c). This is an indication of the prominence of negative pressure events sensed by the transducers when the jet flow impacts the surface.

In order to point out the persistence of these intermittent events, time evolutions of the pressure signals normalized with the signal standard deviation are reported in Fig. 5, versus the adimensional time. As reported in Fig. 5(a), not important intermittent events are detected before the jet impact point. While, more downstream, Fig. 5(b), the pressure time evolution changes its shape and seems more dominated by intermittent positive and negative events larger than 5, these are probably ascribed to both: the jet development and the jet-plate interaction. Finally, the investigation is performed in the zone where there is a slightly developed, Fig. 5(c), and a well developed, Fig. 5(d), TBL-like zone, highlighting the likely presence of the TBL that reduces the existence of positive intermittent events^14^.

In agreement with the *PDF*s shape and time evolutions, downstream of the impact points the skewness factor tends to zero, as an indication of the symmetry of the PDF, and the kurtosis becomes larger than three (Fig. 4b,d) confirming the intermittent dynamics of the pressure events when a TBL-like state is approached. While a negative skewness is detected when there is a developed TBL-like zone.

### Two point statistics

**Figure 7:**
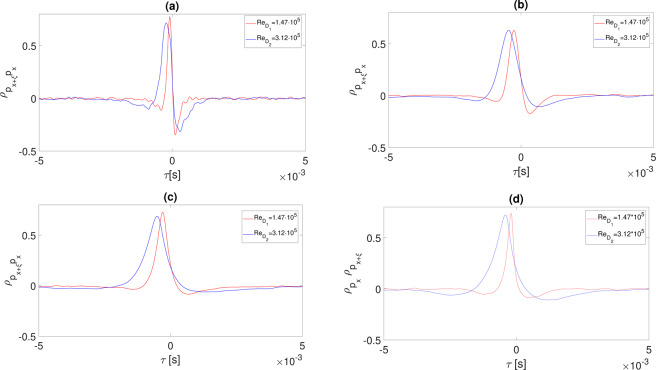
Wall pressure cross-correlation coefficient for different Reynolds numbers, different axial positions *x*/*D* and different plate positions. (**a**) *x*/*D* = 5 and *H*/*D* = 2. (**b**) *x*/*D* = 14 and *H*/*D* = 2. (**c**) *x*/*D* = 23 and *H*/*D* = 2. (**d**) *x*/*D* = 23 and *H*/*D* = 0.75.

The two-point statistics of the wall pressure field at different Reynolds numbers have been computed in the time and frequency domains in terms of cross-correlation and coherence functions. The cross-correlation was computed between consecutive wall pressure signals in the stream-wise direction, as reported in the following:

10 $${R}_{{p}_{x}{p}_{x+\xi }}(x,\xi ,\tau )= < p(x,t)\,p(x+\xi ,t+\tau ) > $$

where *ξ* is the distance between microphones (in the present study *ξ* = 1*D* and it varies according to the nozzle exhaust diameter), *τ* is the time lag and the symbol <> denotes the ensemble average. The cross-correlation coefficient ${\rho }_{{p}_{x}{p}_{x+\xi }}$ is obtained by normalizing the cross-correlation function by the product of the standard deviations of the wall pressure signals. The cross-correlation shows for both Re the typical positive-negative-bump shape associated with the passage of vortical structures^20,23^, as reported in Fig. 7(a). For large *x*/*D* the negative bump disappears and the time scale of the cross-correlations enlarges due to the development of large-scale turbulent structures, Fig. 7(b–d). The increase of the Reynolds number has the effect of broadening the cross-correlations according to the larger scale of the flow structures associated to the larger nozzle diameter. This effect seems to propagate far downstream, as shown in Fig. 7(c,d).

**Figure 8:**
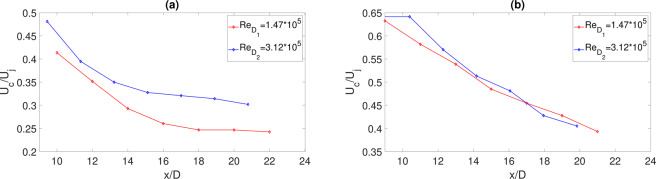
(**a**) Axial evolution of the normalized convection velocity *U*_*c*_ for *H*/*D* = 0.2 (**a**) and *H*/*D* = 0.75 (**b**).

Cross-correlations were exploited to evaluate the convection velocity (*U*_*c*_) along the jet axis. *U*_*c*_ is determined by the ratio between the microphone separation *ξ* and the time lag at which the cross-correlation maximum is found. The convection velocity *U*_*c*_ normalized by the nozzle exhaust velocity *U*_*j*_ is reported in Fig. 8(a) for *H*/*D* = 2 and in Fig. 8(b) for *H*/*D* = 0.75. For both configurations and both *Re* the convection velocity decreases as the axial distance increases. In the case *H*/*D* = 2 a strong dependence upon *Re* and a non-linear decay law with *x*/*D* is observed. At the lower *H*/*D* the decay law is linear and the dependence upon *Re* is weak. This result seems to indicate that the convection velocity is a non trivial function of *Re*, *x*/*D* and *H*/*D*. These dependencies cannot be further exploited through the present data-base and thus a clearer definition of the function *U*_*c*_(*Re*, *H*/*D*, *x*/*D*) remains a challenge for future investigations.

The computation of *U*_*c*_ is of importance for modelling purposes because it represents a crucial ingredient in the formulations usually adopted to predict the coherence function.

**Figure 9:**
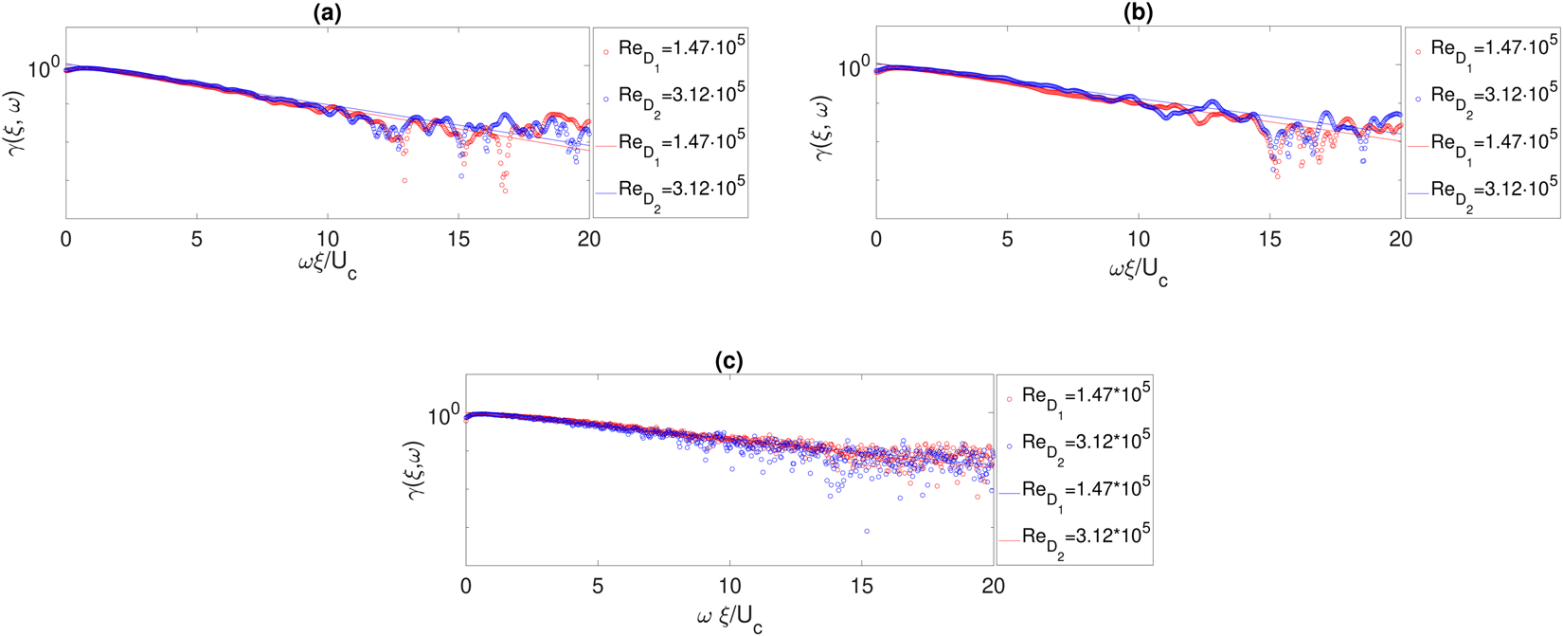
Wall pressure coherence functions for different Reynolds numbers, different axial positions *x*/*D* and different plate positions *H*/*D*. (**a**) *H*/*D* = 2 and *x*/*D* = 19. (**b**) *H*/*D* = 2 and *x*/*D* = 23. (**c**) *H*/*D* = 0.75 and *x*/*D* = 23. Markers are for experimental data and lines for Corcos’ model fit.

The coherence function along the stream-wise direction is evaluated as follows,^19^:

11 $$\gamma (\xi ,\omega )=\frac{|{\Phi }_{p1p2}\,(\xi ,\omega )|}{{[{\Phi }_{p1}(\omega ){\Phi }_{p2}(\omega )]}^{1/2}}$$

where *ω* is the angular frequency, Φ_*p*1*p*2_ the cross-spectrum, Φ_*p*1_ and Φ_*p*2_ the auto-spectra of two consecutive microphones separated in the stream-wise direction by *ξ* = 1*D*. The coherence function was calculated beyond the impact points and explanatory results are reported in Fig. 9. The linear trend in the semi-log representation indicates an exponential decay law that is the expected behavior in fully developed TBLs^13,20^.

No Reynolds effect is detected. Experimental results were compared with the Corcos’ model^29^ that provides a prediction of the coherence function through a simple exponential function:

12 $$\gamma \,(\xi ,\omega )={\exp }\left(-\alpha \frac{\xi \omega }{{U}_{c}}\right)$$

**Figure 10:**
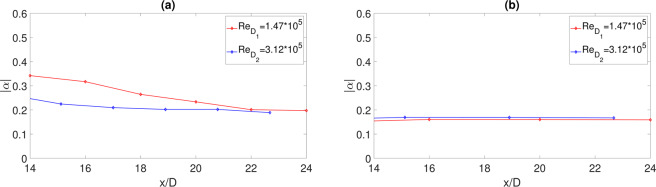
(**a**) Axial evolution of the Corcos’ coefficient for different Reynolds numbers at *H*/*D* = 2. (**b**) Axial evolution of the Corcos’ coefficient for different Reynolds numbers at *H*/*D* = 0.75.

The fits obtained using the Corcos formulation are also reported in Fig. 9 showing a good agreement except for very high frequencies (approximately for $\frac{\omega \xi }{{U}_{c}} > 10$). As expected, the model approximation improves as the distance from the impact point increases. The coefficient *α* has been determined through a least-square algorithm and its evolution in terms of *x*/*D* is presented in Fig. 10. The dependence upon *x*/*D* is weak and, as the axial distance increases, the amplitude tends to a constant value very close to the one expected in a canonical TBL (see, among many^13,29,30^). Also the effect of the jet Reynolds number is not relevant, being negligible for the case at *H*/*D* = 0.75 and weak for *H*/*D* = 2. It can be concluded that downstream of the impact point the coherence function has a quasi-universal shape. It is almost independent of the governing non-dimensional parameters and the Corcos’ model can be reasonably adopted to provide its analytical approximation with a good accuracy.

## Conclusions

The effect of the nozzle-diameter-based Reynolds number on the wall pressure fluctuations induced by a subsonic jet on a tangential flat plate, was investigated at a fixed Mach number. Two Reynolds numbers are achieved by installing on the same air supply system and plenum chamber, two nozzles with different exhaust diameters. The flow is turbulent in both cases (*Re* > 10^5^) the *Re* amplitude is doubled while the Mach number is maintained constant. Wall pressure fluctuations were acquired in the stream-wise direction by means of a couple of pressure transducers flush-mounted on the surface of the tangential plate. The acquired signals were analyzed both in the physical and the Fourier domain and the dependence upon the main non-dimensional parameters has been investigated.

The statistical analysis highlights the effect of the Reynolds number that is partially compensated through proper scaling relationships. A good collapse is achieved in terms of power spectra for which, depending on the region considered, different scaling laws are proposed. A weak dependence upon *Re* is observed for the PDFs and for the corresponding high order moments whereas the cross-correlations shape depends upon *Re* since it tends to broaden for increasing *Re* as an effect of the increased size of the large scale structures. On the other hand, the coherence function, in the region downstream of the impact point, is weakly influenced by *Re* and by the other parameters. The dependence upon the non-dimensional frequency is well represented by an exponential decay law and the simple approach given by the Corcos’ model appears to provide a sufficiently accurate approximation. A critical issue concerns the convection velocity *U*_*c*_ whose dependency upon the non dimensional parameters, *Re* included, seems quite complex and could not be definitely clarified with the present measurements. This aspect surely deserve further investigations. Anyway results provided using different nozzle *Re* numbers are an important basis to improve the physical understanding about possible localized mass flow rate blockage present in full scale jet-wing configurations, like the presence of the plug in the primary nozzle of coaxial jets or the pylon used to fix the jet under the wing.
